# Sexual Dimorphism in Changes That Occur in Tissues, Organs and Plasma during the Early Stages of Obesity Development

**DOI:** 10.3390/biology10080717

**Published:** 2021-07-28

**Authors:** Priyanka Dhanraj, Marlene B. van Heerden, Michael S. Pepper, Melvin A. Ambele

**Affiliations:** 1 Institute for Cellular and Molecular Medicine, Department of Immunology and SAMRC Extramural Unit for Stem Cell Research and Therapy, Faculty of Health Sciences, University of Pretoria, Pretoria 0001, South Africa; priyanka.dhanraj162@gmail.com (P.D.); michael.pepper@up.ac.za (M.S.P.); 2 Department of Oral Pathology and Oral Biology, School of Dentistry, Faculty of Health Sciences, University of Pretoria, Pretoria 0001, South Africa; marlene.vanheerden@up.ac.za

**Keywords:** obesity, adipose tissue, macrophage infiltration, histology, cytokines, sex differences

## Abstract

**Simple Summary:**

Obesity is a global health concern with numerous associated comorbidities. This study aims to provide a qualitative assessment of changes that may occur in tissues, organs, and plasma during the early stages of obesity development and how it may differ between male and female using a mouse model of diet induced obesity. Notable changes, not previously reported, were observed in the lungs, liver, kidney, spleen, and heart, which may suggest early signs of developing an obesity associated comorbidity. Leptin levels with notable sexual dimorphisms changes significantly in early obesity and was observed to also correlate with insulin levels. Interestingly, males and females showed different inflammatory cytokine profiles with females exhibiting a more anti-inflammatory cytokine profile, notably the IL-6/IL-10 axis of cytokine regulation may account for their significantly lower weight gain compared to males. Thus, this study provides valuable information which may aid in understanding the development of some obesity associated diseases at the early stages and could assist in developing effective intervention strategies in males and females.

**Abstract:**

Despite obesity being a major health concern, information on the early clinical changes that occur in plasma and tissues during obesity development and the influence of sexual dimorphism is lacking. This study investigated changes in tissue and organ histology, macrophage infiltration, plasma hormones, lipid, and chemokine and cytokine levels in mice fed on a high fat diet for 11-weeks. An increase in adiposity, accompanied by adipocyte hypertrophy and macrophage infiltration, was observed to be significantly greater in males than females. Important changes in cell morphology and histology were noted in the lungs, liver, kidney, spleen, and heart, which may indicate early signs for developing obesity associated comorbidities. Leptin, but not adiponectin, was significantly altered during weight gain. Additionally, leptin, but not adiposity, correlated with insulin levels. Interestingly, GM-CSF, TNFα, and IL-12 (p70) were not produced in the early stages of obesity development. Meanwhile, the production of MCP-1, IP-10, RANTES, IL-10, IL-6, KC, and IL-9 were greatly influenced by sexual dimorphism. Importantly, IL-6/IL-10 axis of anti-inflammatory cytokine regulation was observed only in females and may account for their significantly lower weight gain compared to males. This study provides new knowledge on how sexual dimorphism may influence the development of obesity and associated comorbidities.

## 1. Introduction

Obesity is characterized by the accumulation of excess body fat or adiposity due to adipocyte hyperplasia (increase in number) or hypertrophy (increase in size). Obesity is tightly associated with metabolic abnormalities such as prediabetes, insulin resistance, high plasma triglyceride, low high-density lipoprotein-cholesterol, and non-alcoholic fatty liver disease, amongst others. The distribution of adipose tissue in obesity has a major impact on human health due to its association with numerous comorbidities, such as type 2 diabetes and cardiovascular disease, as well as reduced life expectancy and increased risk of morbidity and mortality [1,2,3]. Adipose tissue expansion through adipocyte hypertrophy leads to a dysfunctional adipose tissue commonly found in obese individuals [4], which results to adipokine production, adipocyte insulin resistance, macrophage infiltration, and production of pro-inflammatory factors, as well as release of free-fatty acids (FFA) [5]. 

Macrophage infiltration is a key feature of adipose tissue dysfunction and occurs in response to adipocyte necrosis to phagocytose dead or necrosed adipocytes [6,7]. Adipose tissue hypoxia and the release of FFA that occur during adipose tissue expansion may also initiate the recruitment of macrophages. These macrophages are of the M1 phenotype and secrete pro-inflammatory cytokines and chemokines, which further recruits more macrophages and other immune cells into the adipose tissue [6,7]. As a result, obesity is associated with a low-grade chronic inflammation characterized by an increase in proinflammatory cytokines (IL-6, TNF-α, IL-1α) and a reduction in anti-inflammatory cytokines (IL-4, IL-10, and IL-13) [8].

Studies aimed at identifying possible interventions or treatment strategies for obesity have made use of diet-induced obesity (DIO) models such as the C57BL/6J mouse model, which has been used in numerous obesity-related studies [9,10,11]. A majority of these in vivo obesity studies have used male animals to investigate the impact of DIO on adiposity, glucose intolerance, insulin resistance, and inflammation. These studies have largely focused on the chronic or long-term effects of obesity on the different parameters analyzed, including the effect on liver and heart morphology [9,10,11]. Obesity is known to have a major impact on various organ systems that are associated with some of its comorbidities like Type 2 diabetes, cardiovascular disease, pulmonary complications, gallbladder disease, pancreatitis, non-alcoholic fatty liver disease (NAFLD), and cancer [1,8]. However, information on how the cell morphology and histology of various organs and tissues with pathological conditions associated with obesity are affected in the early stages of weight gain and obesity development, is lacking. Furthermore, considering that obesity and the associated comorbidities affect both males and females [12], previous studies have shown bias towards the use of predominantly male animals in investigating these parameters because of concern in the variability associated with the estrous cycle in females [13]. It is, therefore, important for future studies in obesity to utilize both sexes to provide a complete understanding on obesity development and the manifestation of its pathologies, as well as whether sexual dimorphism could be a contributing factor that influences the development of such pathologies.

This study, therefore, used both male and female mouse models of DIO to better understand the influence of sexual dimorphism on obesity development to investigate clinical changes occurring in the cell morphology and histology of various organs and tissues of the endocrine, cardiovascular, respiratory, immune, urinary, and digestive systems in the early stages of weight gain and obesity development. It also evaluated macrophage infiltration into white and brown adipose tissue and further investigated changes in plasma biochemistry, and chemokine and cytokine levels that occur in the early stages of obesity development.

## 2. Materials and Methods

### 2.1. Diet Induced Obesity Model and Study Design

This study was approved by Faculty of Health Sciences Research Ethics Committee and the Animal Ethics Committee, University of Pretoria (protocol no. 209/2019 and 379/2020). The study used 14–17-week-old male and female C57BL/6J mice from The Jackson Laboratory (Bar Harbour, ME, USA). A total of 32 mice (14 males and 18 females) were used. The mice were housed in the animal unit at the Onderstepoort Veterinary Animal Research Unit (OVARU), University of Pretoria, that was maintained at a temperature of 22 °C (±2 °C), relative humidity of 50% (±20%) and a 12-h light–dark cycle. Mice were randomly divided into two experimental groups (16 mice per group), weighed, and fed on either a high fat diet (HFD), 60% kcal fat; D12492, or a nutrient matched control diet (CD), 10% kcal fat; D12450J obtained from Research Diets (New Brunswick, NJ, USA) for a duration of 11 weeks. Measurements of body weight, and food and calorie intake were obtained weekly. 

### 2.2. Intraperitoneal Glucose Tolerance Test

An intraperitoneal glucose tolerance test (GTT) was performed in mice at week 3 and 9. Mice were fasted for 4 h prior to an intraperitoneal administration of 1.5 mg/g body weight glucose solution (Sigma-Aldrich, St. Louis, MO, USA). Baseline measurement of glucose concentration was completed prior to intraperitoneal administration of glucose (0 min) and at 15, 30, 60, 90, and 120 min after administration of glucose. Blood samples were collected by a needle prick of the tail vein at each time point to determine glucose concentration (mmol/L) using a glucometer (Accu-Chek^®^, Roche Diabetes Care, Pretoria, South Africa). 

### 2.3. Organ and Tissue Harvesting

Mice were terminated by isoflurane overdose and blood was collected in EDTA tubes via cardiac puncture during the termination procedure for plasma isolation. The following organs and tissues were harvested from each mouse upon termination: white adipose tissue from the posterior subcutaneous depot (inguinal region) and perigonadal visceral depot, interscapular brown adipose tissue, adrenal glands, heart, spleen, thymus, lung, kidneys, liver, pancreas, and gastrocnemius muscle. All organs and tissues collected were weighed and processed for light microscopy (hematoxylin and eosin) and immunohistochemistry.

### 2.4. Cell Morphology and Histology of Organs and Tissues

Harvested organs and tissues were immediately placed in 10% neutral buffered formalin solution for processing following a standard protocol which involves embedding the organs and tissues in paraffin (formalin fixed paraffin embedded; FFPE), after which 3 µm thick sections were made and mounted on microscope slides. The slides were stained with hematoxylin and eosin using an automatic slide stainer (Leica, Microsystems, Newcastle, UK), and the sections were viewed using a Zeiss AXIO Imager M2 light microscope (Carl Zeiss Microscopy, Munich, Germany).

### 2.5. Immunohistochemistry

FFPE sections were deparaffinized using xylene following an overnight incubation at 54 °C. The sections were then rehydrated through a series of decreasing EtOH concentrations and washed with distilled water. The sections were treated with 3% (*w*/*v*) hydrogen peroxide (H_2_O_2_; Sigma-Aldrich, St. Louis, MO, USA) in methanol for 5 min at 37 °C to block endogenous peroxidase activity. Antigen retrieval with citrate buffer (pH 6.1; Dako Target Retrieval Solution S1699, Dako, Carpinteria, CA, USA) was then performed using a heat induced retrieval method in a Retriever unit 2100 (Electron Microscopy Sciences, Hatfield, PA, USA). Phosphate buffered saline (PBS)/Tween buffer (Sigma-Aldrich, St. Louis, MO, USA) was used to wash the sections followed by incubation with 5% goat serum (Dako X0907, Dako, Carpinteria, CA, USA) at room temperature (RT) for 30 min. The samples were stained with F4/80 monoclonal rat anti-mouse antibody (BM8) (eBioscienceTM, San Diego, CA, USA) at 1:100 dilution and incubated overnight at 4 °C in a humid chamber. The stained sections were washed with PBS/Tween, followed by a 30 min incubation (RT) with goat anti-rat IgG (H+L) secondary antibody, horseradish peroxidase (HRP) at a 1:200 dilution (eBioscienceTM, San Diego, CA, USA). The stained sections were washed and developed with 3,3′-diaminobenzidine (DAB)/HRP development reagent for 15 min. The sections were then counterstained with hematoxylin and washed followed by dehydration through a graded series of EtOH, and then xylene. The slides were mounted with DPX permanent mounting medium. The sections were viewed using a Zeiss AXIO Imager M2 light microscope. Mouse liver was used as the positive control, while the negative control was PBS buffer in place of the antibody.

### 2.6. Measurements of Plasma Hormones and Lipid

Plasma leptin, adiponectin, cholesterol, and insulin were measured in both CD and HFD mice using the following commercially available kits; the Quantikine^®^ ELISA mouse Leptin Immunoassay kit (Catalog No: MOB00; R & D Systems, Minneapolis, MN, USA), the Quantikine^®^ ELISA mouse Adiponectin/Acrp30 Immunoassay kit (Catalog No: MRP300, R & D systems, Minneapolis, MN, USA), cholesterol assay kit (Catalog No: ab65390; Abcam, Cambridge, MA, USA) and mouse insulin (INS) ELISA kit (Catalog No: E-EL-M1382; Elabscience^®^, Houston, TX, USA), respectively, following the manufacturer’s protocol. A microplate scanning spectrometer (BioTek instruments Inc., Highland, CA, USA) was used to measure the optical density for the leptin (450 nm, wavelength correction: 540 nm–570 nm), adiponectin (450 nm, wavelength correction: 540 nm–570 nm) and insulin (450 nm) assays. A fluorometric detection method was used to measure total cholesterol, and optical density (570 nm) was measured on a luminescence spectrometer.

### 2.7. Plasma Cytokine Analysis

The Milliplex^®^ MAP Mouse Cytokine/Chemokine Magnetic Kit 25 plex (Cat: MCYTOMAG-70K-PMX; Merck, Darmstadt, Germany) was used according to the manufacturer’s protocol to measure the following 25 chemokines and cytokines: granulocyte colony stimulating factor (G-CSF), granulocyte-macrophage colony stimulating factor (GM-CSF), interferon gamma (IFN-γ), interleukin 1 alpha (IL-1α), IL-1β, IL-2, IL-4, IL-5, IL-6, IL-7, IL-9, IL-10, IL-12 (p40), IL-12 (p70), IL-13, IL-15, IL-17, interferon- γ inducible protein 10 (IP-10), keratinocyte chemoattractant (KC), monocyte chemoattractant protein 1 (MCP-1), macrophage inflammatory protein 1 alpha (MIP-1α), macrophage inflammatory protein 1 beta (MIP-1β), macrophage inflammatory protein 2 (MIP-2), regulated on activation normal T cell expressed and secreted (RANTES) and tumor necrosis factor alpha (TNF-α). Standards (2000 pg/mL, 400 pg/mL, 80 pg/mL, 16 pg/mL, and 3.2 pg/mL) and controls were prepared as described in the manufacturer’s protocol and transferred into separate wells on a 96 well plate. Samples were diluted with Assay buffer at a 1:2 ratio. Serum matrix was added to the background, standards, and control wells. To the sample wells, 25 µL of Assay buffer was added before the addition of the diluted samples. Premixed cytokine and chemokine antibody-immobilized magnetic beads were added and incubated overnight. The following day, the plate was washed using wash buffers supplied in the kit. Detection antibodies were added, and the plate was incubated for 1 h at room temperature. Streptavidin-phycoerythrin was added to each well and the plate was incubated for 30 min at RT. The plate was washed, and the beads were resuspended in sheath fluid. The plate was read on the Bio-Plex™ 200 system (Bio-Rad, Johannesburg, South Africa). A standard curve was plotted, and the concentrations of chemokines and cytokines were determined or inferred from optical density values and compared between CD and HFD groups.

### 2.8. Statistical Analysis

Results are expressed either as mean ± standard deviation (SD) or mean ± standard error of the mean (SEM). Statistical analyses of measurements between the HFD and CD groups, as well as between males and females, were performed using GraphPad Prism version 7 (GraphPad Software Inc., San Diego, CA, USA). A two-tailed unpaired Student’s *t*-test was used to determine the difference between means. Two-way ANOVA was used to perform multiple comparison within groups followed by the Bonferroni post-test. Differences between means were considered statistically significant at *p* < 0.05. A qualitative analysis of the histology of organs and tissues were performed by comparing the CD and HFD groups. Quantitative analysis of the organ and tissue sections was carried out using image analysis program, ImageJ 1.52v, Java (http://imagej.nih.gov/ij; accessed on 14 October 2020). ImageJ was used to measure cell number, cross sectional area, as well as quantifying macrophage infiltration by measuring the positively stained area per high power field (n = 5 high power field per sample). 

## 3. Results

### 3.1. Males Are More Susceptible to DIO and Showed High Level of Glucose Intolerance Compared to Females

**Figure 1:**
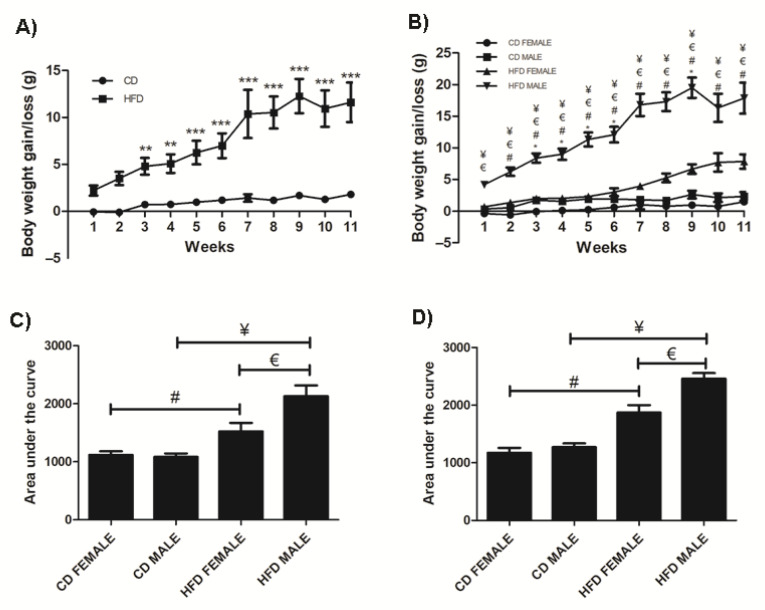
Weight gained in CD and HFD animals over the 11-week period. HFD animals gained significantly more weight than CD animals, (**A**). HFD females and males gained significantly more weight than CD females and males, respectively. Additionally, HFD males gained significantly more weight than HFD females, (**B**). The area under the curve for a glucose tolerance test conducted at week 3, (**C**) and week 9, (**D**) showed HFD males and females displayed glucose intolerance, which was greater in the HFD males. Key: ** Significant difference between CD and HFD animals, *p* < 0.05. *** Significant difference between CD and HFD animals, *p* < 0.001.* Significant difference between CD male and CD female, *p* < 0.05. # Significant difference between CD female and HFD female, *p* < 0.05. ¥ Significant difference between CD male and HFD male, *p* < 0.05. € Significant difference between HFD female and HFD male, *p* < 0.05.

Over the 11-week period, mice on HFD gained significantly more weight (*p* < 0.0001) compared to those on CD starting from week 3 (Figure 1A) and this was observed in both sexes (Females: *p* = 0.0004; Males: *p* < 0.0001) (Figure 1B). The average total weight gained on HFD was 7.86 g ± 1.14 (37.85% ± 4.913) and 17.86 g ± 2.446 (73.04% ± 6.386) in females and males, respectively. Interestingly, males gained significantly more weight than females on both CD and HFD (CD: *p* = 0.0003 and HFD: *p* < 0.0001), which was evident with a notably larger phenotype and fat accumulation than in females (Supplementary Figure S1). Average weekly food intake was similar in males and females on either CD or HFD; however, the caloric intake was significantly higher in the HFD animals (females: *p* = <0.0001; males: *p* < 0.0002) (Supplementary Table S1). Importantly, HFD males gained significantly (*p* = 0.0124) more weight than HFD females (Figure 1B) despite no significant difference in their caloric intake (Supplementary Table S1). Furthermore, the weight gained in HFD males was accompanied by a significantly greater mass of inguinal and perigonadal WAT (iWAT, *p* = 0.007; pWAT, *p* = 0.038) as well as liver mass (*p* < 0.01) compared to HFD females, while there was no difference in the weight of all the other organs (data not shown). Additionally, the glucose tolerance in animals at week 3 and week 9 were similar, however, HFD animals displayed greater levels of glucose intolerance compared to the CD animals and this was more evident in males than females at both week 3 and week 9 (Figure 1C,D).

### 3.2. Significant Changes in Cell Morphology and Histology Occur Early in Obesity Development

**Figure 2:**
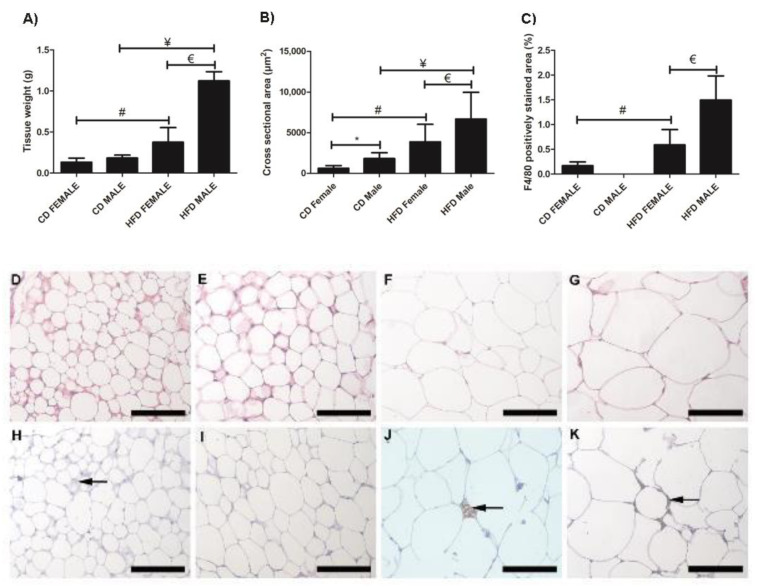
Histology and immunohistochemistry of iWAT sections. Weight of iWAT sections, (**A**); cross sectional area of adipocytes, (**B**); and F4/80+ macrophage-stained area, (**C**). Histological sections showing adipocyte sizes in CD females, (**D**); CD males, (**E**); HFD females, (**F**); and HFD males, (**G**). Adipocyte hypertrophy was evident in HFD males and females, compared to the CD males and females. F4/80+ immunostained sections from the CD females, (**H**); CD males, (**I**); HFD females, (**J**); and HFD males, (**K**). Macrophage infiltration (black arrow) was evident in CD females, (**H**); HFD females, (**J**); and HFD males, (**K**). No macrophages were observed in CD males, (**I**). Significantly greater macrophage infiltration was observed in HFD animals. * Significant difference between CD females and CD males, *p* < 0.05. # Significant difference between CD female and HFD female, *p* < 0.05. ¥ Significant difference between CD male and HFD male *p* < 0.05. € Significant difference between HFD female and HFD male, *p* < 0.05. Mean ± SD. Scale bar: 100 µm.

**Figure 3:**
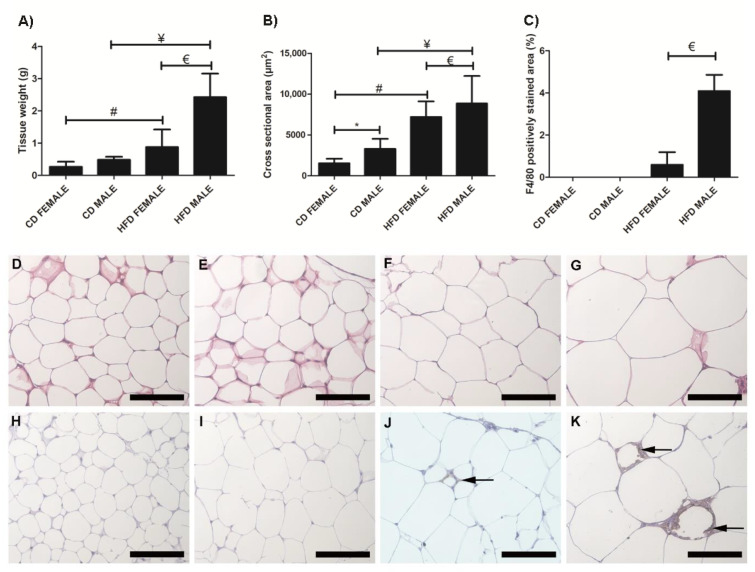
Histology and immunohistochemistry of pWAT sections. Weight of pWAT sections, (**A**); cross sectional area of adipocytes, (**B**); and F4/80 macrophage-stained area, (**C**). Adipocyte hypertrophy was evident in HFD males, (**G**) and females, (**F**) compared to CD males, (**E**) and female, (**D**), respectively. F4/80+ immunostained sections from the CD females, (**H**); CD males, (**I**); HFD females, (**J**); and HFD males, (**K**). Macrophage infiltration (black arrow) was evident only in the HFD females, (**J**); and males, (**K**); no macrophages were observed in the CD females, (**H**); and males, (**I**). A significantly greater macrophage infiltration was observed in the HFD males compared to HFD females, (**C**). * Significant difference between the CD females and CD males, *p* < 0.05. # Significant difference between CD female and HFD female, *p* < 0.05. ¥ Significant difference between CD male and HFD male *p* < 0.05. € Significant difference between HFD female and HFD male, *p* < 0.05. Mean ± SD. Scale bar: 100 µm.

**Figure 4:**
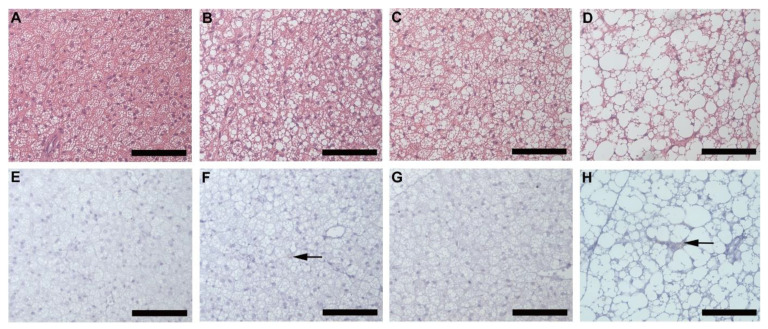

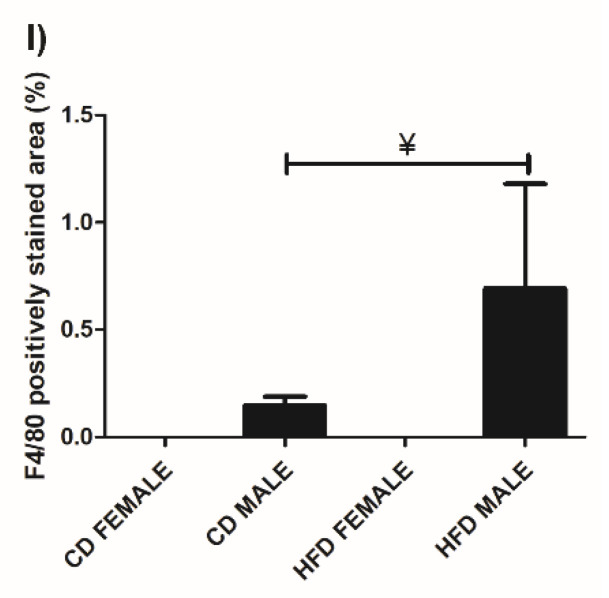
Histology and immunohistochemistry of BAT. Histological sections showing adipocytes in BAT of CD females, (**A**); CD males, (**B**); HFD females, (**C**); and HFD males, (**D**). Adipocyte expansion was evident in the HFD males, (**D**) and females, (**C**) compared to the CD males, (**B**) and females, (**A**). F4/80+ immunostained sections from CD females, (**E**); CD males, (**F**); HFD females, (**G**); and HFD males, (**H**). Macrophage infiltration (black arrow) was only evident in males on CD, (**F**) and HFD, (**H**) with the HFD males displaying a significantly greater extent of macrophage infiltration, (**I**). No macrophages were observed in the females on either CD, (**E**) or HFD, (**G**). ¥ Significant difference between CD male and HFD male *p* < 0.05. Mean ± SD. Scale bar: 100 µm.

Inguinal white adipose tissue (iWAT) from HFD animals was significantly larger (Figure 2A) in both males (*p* = 0.018) and females (*p* = 0.0002), compared to their control counterparts. Both HFD females (Figure 2B,F) and males (Figure 2B,G), showed significant (*p* = 0.0001) adipocyte hypertrophy compared to the CD females (Figure 2B,D) and males (Figure 2B,E). Interestingly, the males displayed significantly greater adiposity (*p* = 0.001) and adipocyte hypertrophy (*p* = 0.0001) than females on HFD, while on CD, males displayed significantly larger (*p* = 0.0001) adipocytes than females (Figure 2B). Adipocyte hypertrophy in HFD animals was accompanied by a significant increase in macrophage infiltration in adipose tissues, especially in males (Figure 2C,H–K). Similar observations were made in perigonadal adipose tissue (pWAT), with greater adiposity (Figure 3A, Females: *p* = 0.043; Males: *p* = 0.01) and adipocyte hypertrophy (Figure 3B,D–G; *p* < 0.0001) in HFD animals, especially in HFD males. Macrophage infiltration in pWAT was only observed in HFD animals, and was significantly greater (*p* < 0.0001) in males (Figure 3C,H–K). Adipocyte expansion was also evident in the interscapular BAT (Figure 4A–D) of HFD animals, with HFD males displaying a greater extent of adipocyte expansion (Figure 4D). Interestingly, macrophage infiltration was only observed in males of both experimental groups (Figure 4F,H) and was significantly greater (*p* = 0.0036) in HFD males (Figure 4I,E–H).

**Figure 5:**
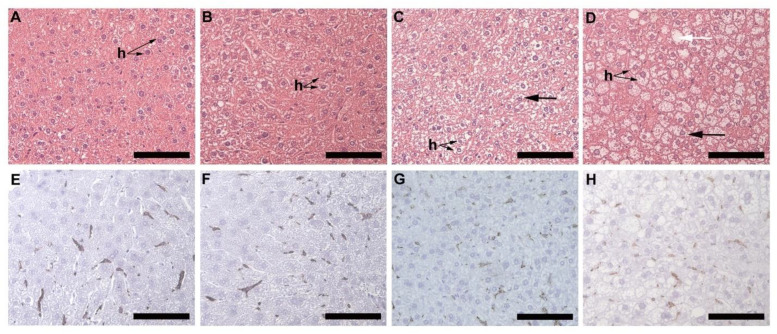

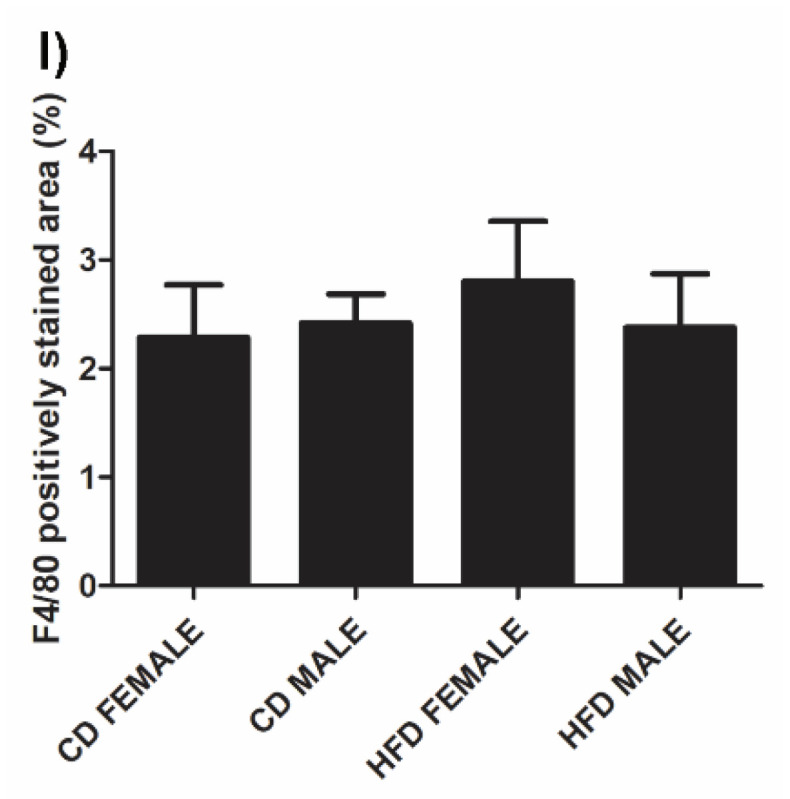
Histology and immunohistochemistry of liver tissues. Histology of liver sections harvested from CD females, (**A**); CD males, (**B**); HFD females, (**C**); and HFD males, (**D**). Microvesicular steatosis (black arrow) characterized by the accumulation of lipid droplets in the hepatocytes (h) was evident in the HFD animals (**C**,**D**), more prominently in the HFD males (**D**) with larger lipid vacuoles (white arrow) present. F4/80+ immunostained sections from the CD females, (**E**); CD males, (**F**); HFD females, (**G**); and HFD males, (**H**); showed positive staining (black arrow) in all groups, with no significant difference being observed; (**I**). Mean ± SD. Scale bar: 100 µm.

Microvesicular liver steatosis presenting as small lipid vacuoles (black arrow) lying within the hepatocytes (Figure 5A–D) was evident in the liver of HFD females (Figure 5C) and males (Figure 5D), but not in CD females (Figure 5A) and males (Figure 5B). In the HFD group, the males presented with a greater degree of steatosis, with larger lipid vacuoles (white arrow) diffused throughout the sections (Figure 5D). The presence of macrophages was observed in all animals, CD females (Figure 5E), CD males (Figure 5F), HFD females (Figure 5G) and HFD males (Figure 5H) with no significant difference observed across all the experimental groups (Figure 5I).

**Figure 6:**
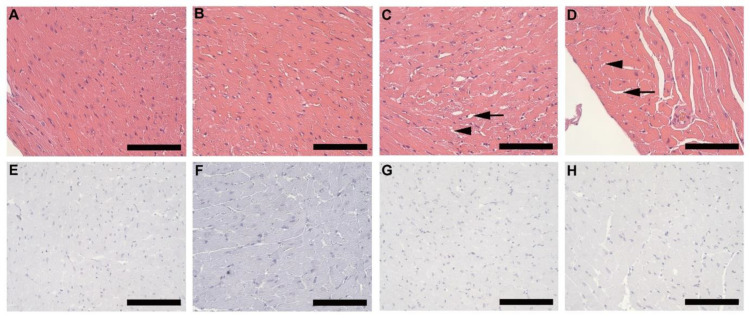
Histology and immunohistochemistry of heart tissue. Histology of heart tissue from CD females, (**A**); CD males, (**B**); HFD females, (**C**); and HFD males, (**D**); showed enlarged myocardial fibers and surrounding fibrocollagenous tissue (black arrow) in HFD animals as well as lipid droplets (arrowhead) that lies between the myocardial fibers in the HFD animals. These features were more prominent in HFD males. F4/80+ immunostained sections from CD females, (**E**); CD males, (**F**); HFD females, (**G**); and HFD males, (**H**); showed the absence of macrophages in heart tissues of all experimental groups. Scale bar: 100 µm.

Alterations such as partial enlargement of myocardial fibers and of surrounding fibrocollagenous tissue (black arrow), as well as visible lipid droplets (arrowhead) lying between the myocardial fibers was evident in both HFD females (Figure 6C) and males (Figure 6D), but not in CD females (Figure 6A) and males (Figure 6B). The alterations observed in HFD animals was greater in males than females. No macrophages were detected in any of the experimental groups (Figure 6E–H).

**Figure 7:**
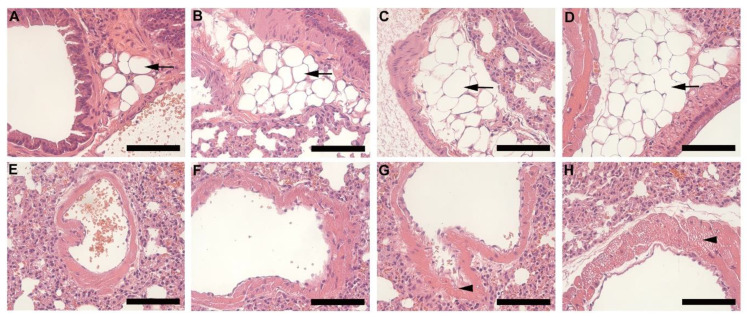
Histology of lung tissue harvested from animals on CD and HFD. CD females, (**A**); CD males, (**B**); HFD females, (**C**); and HFD males, (**D**); illustrates the pulmonary vasculature (bronchiole, pulmonary artery, or vein) surrounding adipocytes (black arrow). Adipocytes were larger and more prominent in the HFD groups (**C**,**D**) than CD (**A**,**B**). The pulmonary artery of CD females, (**E**); CD males, (**F**); HFD females, (**G**); and HFD females, (**H**); shows lipid accumulation (arrowhead) in the smooth muscle layer, which was evident in the HFD groups (**G**,**H**), particularly in HFD males, (**H**). Scale bar: 100 µm.

Histology of lung tissue revealed the presence of adipocytes (black arrow) surrounding the pulmonary vasculature in all animals (Figure 7A–D), however, this was more prominent and covered a larger section of tissue in the HFD groups (Figure 7C,D). In HFD animals, lipid accumulation (arrowhead) was also observed in the smooth muscle layer of the pulmonary artery (Figure 7E–H). This was prominent and visible in HFD females (Figure 7G) and males (Figure 7H) but more so in males.

**Figure 8:**
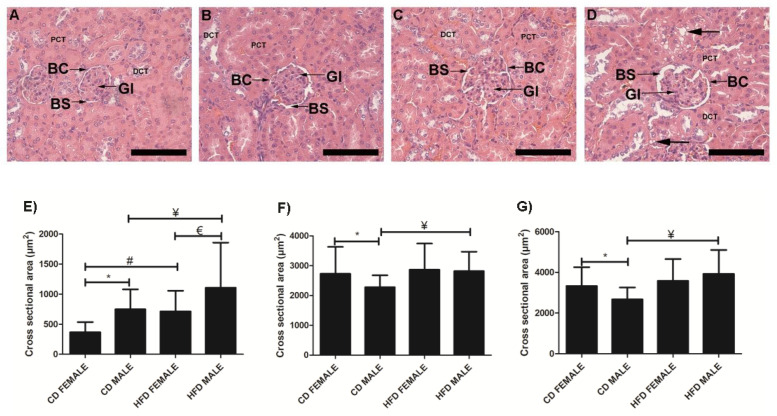
Histology of kidney tissue harvested from CD females, (**A**); CD males, (**B**); HFD females, (**C**); and HFD males, (**D**); showing the glomeruli (Gl), Bowman’s space (BS), Bowman’s capsule (BC), surrounding proximal convoluted tubules (PCT) and distal convoluted tubules (DCT). Cross-sectional area of Bowman’s space, (**E**); glomeruli, (**F**); Bowman’s capsule, (**G**); measured from CD females, CD males, HFD females, and HFD males. The HFD females displayed a significantly enlarged Bowman’s space compared to CD females, E while HFD males displayed significantly enlarged glomerular area, F Bowman’s space, E and Bowman’s capsule, G compared to CD males. Lipid accumulation (black arrow) in the lining and wall of the renal tubule was evident in HFD males, (**D**). * Significant difference between CD females and CD males. # Significant difference between CD female and HFD female. ¥ Significant difference between CD male and HFD male. € Significant difference between HFD female and HFD male. *p* < 0.05. Mean ± SD. Scale bar: 100 µm.

Kidney tissues from CD females (Figure 8A), CD males (Figure 8B), HFD females (Figure 8C) and HFD males (Figure 8D) showed significant enlargement of the Bowman’s space (Figure 8E) in both HFD females (*p* < 0.0001) and HFD males (*p* = 0.0009), while significant enlargement of the glomerular area (*p* = 0.033; Figure 8F) and Bowman’s capsule (*p* < 0.0001; Figure 8G) was only evident in HFD males. Lipid accumulation in the lining and wall of the renal tubules (black arrow) was clearly visible in HFD males (Figure 8D). It was also noted that CD females displayed a significantly larger glomerular area (*p* = 0.0261; Figure 8F) and Bowman’s capsule (*p* = 0.0041; Figure 8G) compared to CD males, while CD males displayed a significantly larger (*p* < 0.0001) Bowman’s space compared to CD females.

**Figure 9:**
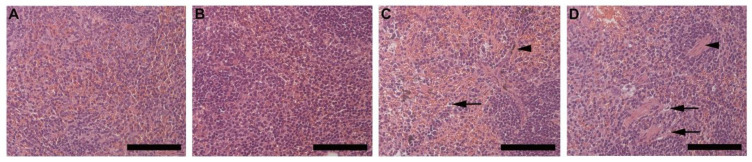
Histology of the spleen harvested from CD females, (**A**); CD males, (**B**); HFD females, (**C**); and HFD males, (**D**) showing the red pulp of the spleen, consisting of the parenchyma and surrounding sinusoids. HFD females, C and males, D displayed cell swelling and necrosis (black arrow), and visible fibrotic lesions (arrowhead). The fibrotic lesions were more prominent and larger in the HFD males. Scale bar: 100 µm.

Analysis of the spleen (red pulp) revealed cell swelling and necrosis (black arrow) in both HFD females (Figure 9C) and males (Figure 9D) but not in CD females (Figure 9A) and males (Figure 9B). Fibrotic lesions (arrowhead) were also observed in HFD animals, with larger and more prominent lesions visible in males than females. In the pancreas, the cross-sectional area of the islets of Langerhans in CD females (Figure S2A), CD males (Figure S2B), HFD females (Figure S2C) and HFD males (Figure S2D) showed that of HFD males was significantly smaller than those in CD males (Figure S2E). Enlargement of the pancreatic acinar cells (black arrow) was also evident in HFD males only (Figure S2D), with partial thickening of the fibrocollagenous septa (arrowhead) (Supplementary Figure S2). Alterations observed in the thymic tissue includes diffused lymphocyte necrosis (arrowhead) and prominent macrophages (black arrow), which were observed in the thymic cortex of HFD animals, particularly in HFD males (Figure S3A–D). Epitheliocyte swelling (white arrow) was also evident in both HFD females (Figure S3G) and males (Figure S3H) but was more prominent in males (Supplementary Figure S3). Alterations to the adrenal glands includes partial enlargement of the cells of the zona fasciculata in HFD animals which was more prominent in males. Chromaffin cells making up the adrenal medulla appeared enlarged in HFD males with a foamy-like appearance (Supplementary Figure S4). Gastrocnemius muscle showed fat accumulation within the perimysium which was observed only in HFD females and males (Supplementary Figure S5). 

### 3.3. Leptin Levels Are Significantly Altered in the Early Stages of Obesity Development

**Figure 10:**
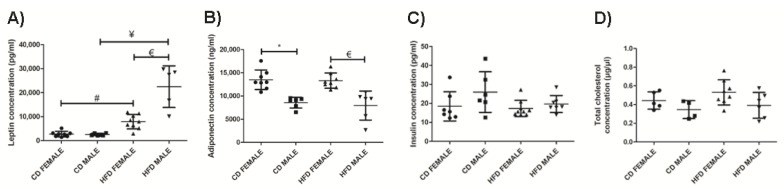
Plasma leptin concentration (pg/mL), (**A**); plasma adiponectin concentration (ng/mL), (**B**); plasma insulin concentration (pg/mL), (**C**); and total cholesterol (µg/µL), (**D**); measured in CD females, CD males, HFD females and HFD males. * Significant difference between the CD females and CD males, *p* < 0.05. # Significant difference between CD female and HFD female, *p* < 0.05. ¥ Significant difference between CD male and HFD male *p* < 0.05. € Significant difference between HFD female and HFD male, *p* < 0.05. Mean ± SD. Black circle, triangle and square represent individual data points in the different categories CD females, CD males, HFD females and HFD males on the x-axis.

Both females and males on HFD displayed a significantly higher plasma leptin concentration compared to females (*p* = 0.0003) and males (*p* = 0.0003) on CD, respectively. HFD males displayed a significantly higher (*p* = 0.0005) plasma leptin concentration than HFD females (Figure 10A). There was no significant change in plasma adiponectin concentration between the HFD and CD animals of both sexes (Figure 10B). However, females displayed significantly higher adiponectin levels than male on either CD or HFD (CD: *p* = 0.0002; HFD: *p* = 0.0013). Measurement of plasma insulin (Figure 10C) and total cholesterol concentration (Figure 10D) revealed minor changes which were not significantly different between the CD and HFD animals of both sexes.

### 3.4. Inflammatory Cytokine Profile in Males and Females Differ in the Early Stages of Obesity Development 

**Figure 11:**
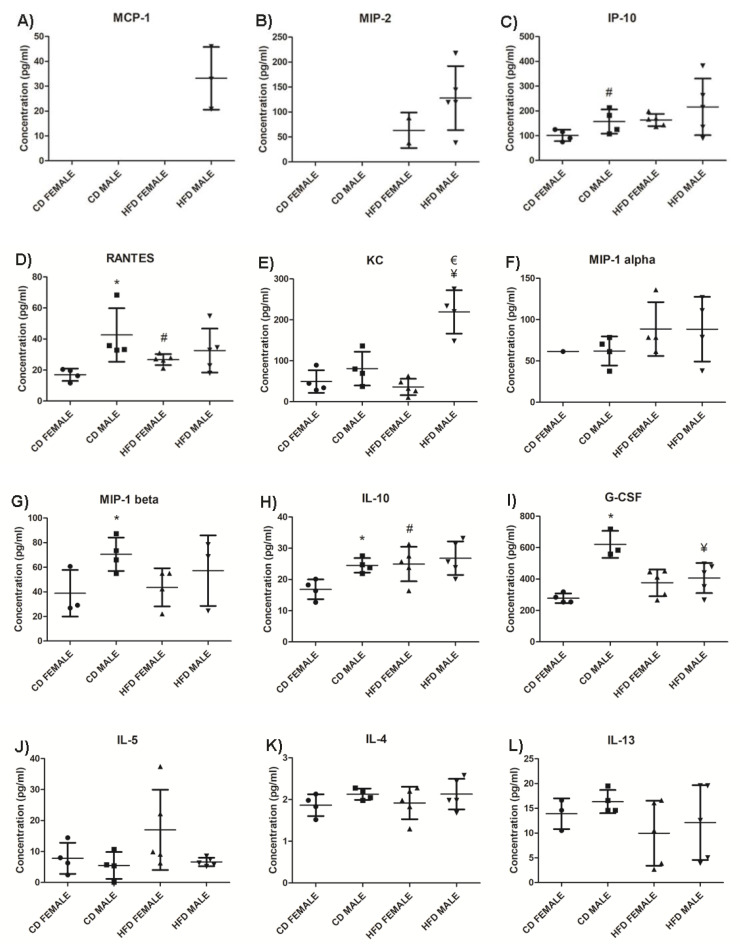

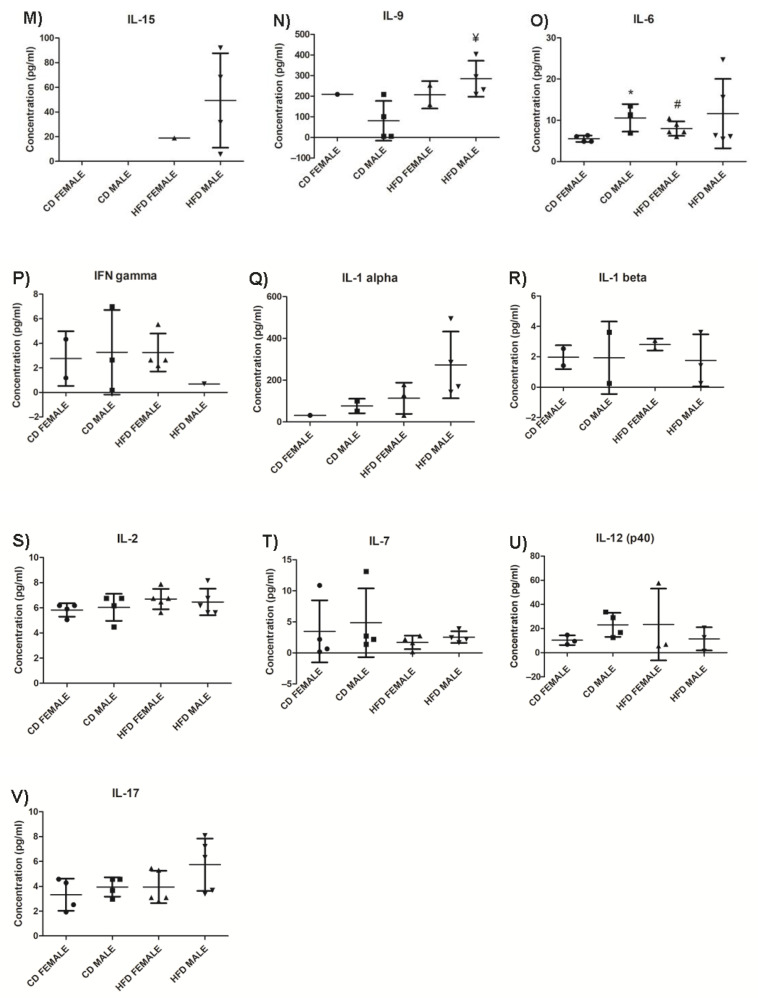
Chemokines, anti-inflammatory and pro-inflammatory cytokines levels in HFD and CD animals. MCP-1, (**A**); MIP-2, (**B**); IP10, (**C**); RANTES, (**D**); KC, (**E**); MIP-1α, (**F**); MIP-1β, (**G**); IL-10, (**H**); G-CSF, (**I**); IL-5, (**J**); IL-4, (**K**); IL-13, (**L**); IL-15, (**M**); IL-9, (**N**); IL-6, (**O**); IFN-γ, (**P**); IL-1α, (**Q**); IL-1β, (**R**); IL-2, (**S**); IL-7, (**T**); IL-12 (p40), (**U**); and IL-17, (**V**). The graphs display the cytokines measured in CD females, CD males, HFD females and HFD males. * Significant difference between the CD females and CD males, *p* < 0.05. # Significant difference between CD female and HFD female, *p* < 0.05. ¥ Significant difference between CD male and HFD male *p* < 0.05. € Significant difference between HFD female and HFD male, *p* < 0.05. Mean ± SD. Black circle, triangle and square represent individual data points in the different categories CD females, CD males, HFD females and HFD males on the x-axis.

The effect of DIO on plasma chemokine and cytokine concentrations was assessed. The chemokines MCP-1 (Figure 11A, Supplementary Table S2) and MIP-2 (Figure 11B, Supplementary Table S2) were only detected in the HFD group, while MCP-1 was only detected in HFD males, MIP-2 was detected in both HFD males and females, with a higher concentration in males. The HFD animals displayed a higher concentration of IP-10, particularly the HFD females which displayed a significantly higher (*p* = 0.006) concentration of IP-10 than their control counterparts (Figure 11C, Supplementary Table S2). Only HFD females displayed a significantly higher (*p* = 0.006) concentration of RANTES than CD females, with males showing no difference between HFD and CD (Figure 11D, Supplementary Table S2). HFD males displayed a significantly higher concentration of KC than HFD females (*p* = 0.0002) as well as when compared to CD males (*p* = 0.006) (Figure 11E, Supplementary Table S2). On average, males displayed higher concentrations of KC than females when on either a CD or HFD. No significant difference was observed in MIP-1α (Figure 11F, Supplementary Table S2) and MIP-1β (Figure 11G, Supplementary Table S2).

From the anti-inflammatory cytokines measured, IL-10 was significantly higher (*p* = 0.046) in HFD animals compared to those on CD, and this difference was only significant in females (*p* = 0.036) (Figure 11H, Supplementary Table S2). Interestingly, CD males showed higher levels of IL-10 than females, which did not change significantly during weight gain on HFD (Supplementary Table S2). G-CSF (Figure 11I, Supplementary Table S2) was significantly lower in HFD males compared to CD males (*p* = 0.0195). Conversely, the concentration of G-CSF in HFD females was higher than in CD females. IL-5 was higher in HFD females than CD females, as well as when compared to HFD males, while there was no difference between the males on CD and HFD (Figure 11J, Supplementary Table S2). No significant difference was observed in IL-4 (Figure 11K, Supplementary Table S2) and IL-13 (Figure 11L, Supplementary Table S2) in both females and males between HFD and CD.

From the pro-inflammatory cytokine profile, IL-15 was detected only in the HFD group and was higher in males (Figure 11M, Supplementary Table S2). Only HFD males displayed a significantly higher (*p* = 0.02) concentration of IL-9 compared to their control counterparts (Figure 11N, Supplementary Table S2). Interestingly, IL-9 expression was infrequent in females of both experimental groups. HFD females displayed a significantly higher (*p* = 0.036) concentration of IL-6 compared to CD females, with no difference being observed between males on HFD and CD (Figure 11O, Supplementary Table S2). It was also noted that, in the CD group, males displayed a significantly higher (*p* = 0.03) baseline concentration of IL-6 compared to females. Insignificant differences were observed in IFN-γ (Figure 11P, Supplementary Table S2), IL-1α (Figure 11Q, Supplementary Table S2), IL-1β (Figure 11R, Supplementary Table S2), IL-2 (Figure 11S, Supplementary Table S2), IL-7 (Figure 11T, Supplementary Table S2), IL-12 (p40) (Figure 11U, Supplementary Table S2), and IL-17 ((Figure 11V, Supplementary Table S2) in both females and males between HFD and CD. GM-CSF, TNFα, and IL-12 (p70) were not detected in either the CD or HFD groups (Supplementary Table S2).

## 4. Discussion

Obesity is a major health concern and is associated with various comorbidities that affect different organ and tissue systems, reducing life expectancy and increasing the risk of morbidity and mortality [1,2,3]. Most studies designed to combat obesity have focused on exploiting the process of fat cell formation (adipogenesis) by identifying important molecular factors which could be used as potential targets to regulate adipogenesis [14,15,16] with the hope of combating obesity. However, not much is known about the changes in plasma concentrations of important mediators, as well as in organs and tissues morphology during the early stages of obesity development which may contributes to defining an obese environment, and whether sexual dimorphism is a contributing factor. This study has, therefore, used male and female C57BL/6J mouse model of DIO to investigate the changes that occur in various organs and tissues, as well as in plasma hormones, lipids, chemokines and cytokines during the initial period of weight gain (11 weeks) leading to obesity development in both males and females. 

The average food consumption per week for females and males on either CD or HFD was similar. However, the caloric intake for both females and males on HFD was significantly higher than their respective counterparts on CD. This translated to a significant weight gain in both females and males on HFD, which is consistent with previous reports [9,11,17,18]. Interestingly, males on HFD gained significantly more weight than females on HFD despite no difference in the amount of food consumed or amount of energy intake. This indicates males are more susceptible to developing DIO than females and suggests energy and fat metabolic processes are influenced by sexual dimorphism, which may account for the significant difference in weight gain between females and males. Other in vivo studies have highlighted that male are more susceptible to DIO and that the effects of DIO persist in females in the later stages [17,19,20,21]. Significant weight gain in HFD animals was associated with increased glucose intolerance at both week 3 and 9. This was more evident in males than females. Increased glucose intolerance or prediabetic conditions are associated with weight gain and obesity, and this study shows the development of this metabolic condition is evident in the early stages of obesity development. 

A significant increase in adipocyte hypertrophy in iWAT and pWAT was evident in HFD animals. This was more evident in males, which displayed significantly larger adiposity and adipocyte sizes than females. Thus, it was clear that DIO leads to adipocyte hypertrophy, which is seen as an adaptive response to excess calorie intake [22]. Adipocyte hypertrophy is a common feature of obesity and is responsible for adipose tissue dysfunction, hypoxia, necrosis, and an inflammatory response, leading to adipokine release and macrophage recruitment [6,7,23]. Increased macrophage infiltration was observed in iWAT, pWAT, and BAT of HFD animals, with males displaying significantly more macrophages, thus indicating a clear association between adipocyte size and macrophage infiltration in obesity development. Adipocyte expansion or “whitening” of the BAT was also observed in HFD animals. This phenomenon was more prominent and evident in the HFD males. Macrophage infiltration was, however, only evident in males and was significantly greater in HFD males. It is not clear why macrophages were only detected in males, but it is clear that BAT “whitening” leads to macrophage infiltration.

Micro-vesicular liver steatosis was observed in HFD animals, more extensively in males, and this was accompanied by a significant increase in liver weight in males compared to females on HFD. An increase in adipocyte hypertrophy may lead to a greater amount of free fatty acids (FFA) being released into circulation, which is taken up by the liver, thereby leading to the steatosis observed. Liver steatosis is commonly associated with obesity, and it is a key feature of non-alcoholic fatty liver disease [24]. Steatosis occurs as a result of greater fatty acid uptake and de novo fatty acid synthesis [25]. It is thus evident that liver steatosis presents in the early stages of weight gain, and further strengthens the link between adipocyte hypertrophy and steatosis in obesity. Assessment of macrophage infiltration as a result of increased steatosis revealed no significant difference between any of the experimental groups, which suggest that either macrophage infiltration does not occur during these early stages or that since macrophages are resident cells of the liver (Kupfer cells), it is macrophage activation rather than infiltration which may occur [26]. This however requires further investigation to determine the proportion of M1/M2 macrophages present in the respective groups.

Signs of glomerular hyperfiltration, such as an increase in glomerular area and volume, and Bowman’s space, were noted in HFD animals. Glomerular hyperfiltration is suggested to be the link between obesity and kidney abnormalities or diseases [27,28]. Additionally, lipid accumulation in the renal tubules was also observed, which has previously been described [27]. Thus, it is evident that important changes occur in kidney structure which may affect its function early in obesity development.

Spleen tissue from HFD animals showed cell swelling and necrosis, and sinusoid dilatation in the red pulp. Fibrotic lesions were also observed in both females and males on HFD but were more prominent and extensive in males. Although these observations are common features of splenomegaly or spleen enlargement [29], a primary feature is an increase in splenic weight which was not observed in the current study. Splenic enlargement is found to persist in the chronic stages of obesity (long-term feeding) [30]. Based on the observations in the current study, it is suggested that the initial effects of DIO on the spleen may include necrosis, sinusoid dilatation and fibrosis, which may later result in splenic enlargement. Intermuscular fat infiltration was observed in the gastrocnemius muscle of HFD animals, and is a common feature in obesity, primarily occurring during adipose tissue expansion [5,7]. This was more evident in males than females on HFD and suggests that this occurs very early in obesity development. Previously reported effects of obesity on the heart include cardiac hypertrophy, lipid accumulation and fibrosis [31]. However, in the current study minor alterations to the heart were observed in HFD animals, which included slight enlargement of myocardial fibers, thickening of the fibrocollagenous tissue and visible lipid vacuoles. No macrophages were detected in the heart tissue, thus suggesting the impact on heart tissue in this regard in the early stages of obesity and weight gain is minimal. The same can be said regarding the pancreas, thymus, and adrenal glands, where only minor alterations were observed. The accumulation of fat in the airways of obese individuals has recently been described [32]. Interestingly, in our study we observed fat accumulation surrounding the pulmonary vasculature and lipid accumulation within the smooth muscle of the pulmonary artery, which to our knowledge has not been described in the context of DIO. The accumulation of fat and lipids within or surrounding the pulmonary vasculature may affect the function of these vessels, leading to pulmonary arterial hypertension, which is a common feature of obesity. Thus, this finding may assist in understanding the development of pulmonary complications associated with obesity. Our study unequivocally shows that DIO caused significant clinical changes in cell morphology and histology of various organs and tissues in HFD animals, which may help in our understanding of the development of some obesity associated pathological conditions.

Changes in plasma adipokine and cytokine levels have been reported in obesity [33]; however, there are a limited number of reports on how these plasma analytes are altered during weight gain and the influence of sexual dimorphism on them. Hyperleptinemia was evident in both HFD females and males, which is consistent with previous studies [33,34,35,36]. Leptin is primarily produced by adipocytes and is proportional to adipose tissue mass, and this was evident in the current study with the HFD males, which displayed significantly higher levels of leptin compared to females. Measurement of adiponectin revealed no significant difference between HFD and CD animals of both sexes. It has been suggested that changes in adiponectin becomes apparent in the later stages of obesity and are largely influenced by insulin resistance [33,37]. Adiponectin expression was also found to be sexually dimorphic, with females displaying a significantly higher concentration than males on either CD or HFD. There was no significant difference in plasma insulin and total cholesterol concentrations between the HFD and CD groups of both sexes, suggesting that significant changes in these analytes may only be apparent in the later stages of obesity. It was noted that plasma insulin levels were lower in animals on HFD compared to those on CD, which is contradictory to many studies that have reported increase insulin levels in obesity [18,38]. Conversely, other studies have reported leptin to reduce circulating glucose and insulin levels, as well as improve insulin sensitivity, and that these actions are independent of body weight [39,40,41]. Additionally, HFD animals have been reported to display significantly higher insulin levels only after 14 weeks on HFD [33], which was not attained in the current study. Therefore, the hypotheses that insulin levels only increase after long-term feeding on HFD (increased adiposity) and that leptin reduces insulin levels, are seemingly possible. We investigated both adiposity and leptin to decipher the relationship between adiposity, leptin, and insulin levels and whether sexual dimorphism plays a role.

Considering adiposity to be a factor that may influence insulin levels, it was observed that HFD males with significantly higher adiposity than CD males did not have higher levels of plasma insulin, as would have been expected should adiposity have been a determining factor at this stage. This suggests that factors other than adiposity may influence plasma insulin levels in periods of early weight gain. Interestingly, when considering leptin as a determinant for insulin levels, CD males displayed significantly lower leptin levels than HFD males, and this was observed to correlate inversely with insulin levels. This important observation suggests that plasma leptin could be a more important factor in regulating insulin levels rather than adiposity per se in the early stages of weight gain, and that this effect is more visible in males than females particularly when on HFD (effect resulting from DIO). Therefore, in the current study, the significantly higher levels of leptin in HFD compared to CD animals may have led to the decrease in plasma insulin levels observed in HFD.

The impact of DIO on chemokine and cytokine concentrations was investigated. MCP-1 and MIP-2 were only detected in the HFD group. MCP-1 is produced by adipocytes, and MIP-2 is produced by macrophages, and elevated levels are associated with obesity [42,43,44]. This indicates the production of MCP-1 and MIP-2 is strongly associated with increased adiposity and weight gain. A higher concentration of IP-10 was observed in HFD animals with only females displaying a significantly higher IP-10 on HFD compared to CD. Similarly, RANTES levels were only significantly elevated in HFD females compared to CD females. This suggest changes in IP-10 and RANTES levels during weight gain are sexually dimorphic and are predominantly evident in females during early stages of obesity development. KC, which is the mouse ortholog of human IL-8, was significantly higher only in HFD males. KC/IL-8 is produced by adipocytes, and elevated levels have been observed in obese mice and humans [45,46]. Thus, significantly high KC/IL-8 observed only in HFD males suggest sexual dimorphism in its production, and that changes in IL-8 during early periods of weight gain is greater in males. 

Of the pro-inflammatory cytokines measured, IL-15 was only detected in the HFD group, indicating an association with body weight, adiposity, and macrophage infiltration. Although there is much controversy regarding the role of IL-15 in obesity [47,48,49,50], our study strongly suggests it plays a role in weight gain and adipose tissue expansion during DIO. IL-9 concentrations were significantly higher in HFD compared to CD animals. There have been reports of elevated levels of IL-9 in the obese microenvironment [51]; however, our study appears to be the first to report an increase in IL-9 expression in response to DIO. Interestingly, it was also noted that HFD males display a significantly higher concentration than CD males, while production of IL-9 was barely seen in females in both experimental groups, suggesting sexual dimorphism influences IL-9 production with dominant expression in males. G-CSF was significantly lower in HFD males than CD males, with no difference observed between the females on the different diets. G-CSF is reported to inhibit and decrease pro-inflammatory cytokines and displays anti-obesity effects by increasing energy expenditure and reducing body weight [52]. The significantly lower concentration of G-CSF observed in HFD males suggests an increased pro-inflammatory cytokine profile and a reduced anti-inflammatory cytokine profile in males.

Of the anti-inflammatory cytokines measured, IL-10 was significantly higher only in HFD females compared to CD females. IL-10 is found to inhibit synthesis and functioning of pro-inflammatory cytokines, as well as suppress the functioning of macrophages [53,54]. This suggests a reduced inflammatory response in adipose tissue in females despite the presence of macrophage infiltration, and a better regulation of pro-inflammatory cytokine production. In addition, IL-6 levels were significantly higher only in females when comparing HFD to CD. IL-6 has been reported as a regulator of energy and glucose metabolism [55], which may contribute to the lower weight gain and lesser degree of glucose intolerance observed in females than males when on HFD for a short period of time. Conversely, despite IL-6 being a pro-inflammatory cytokine, it has also been reported to exhibit anti-inflammatory properties [56,57]. One way in which IL-6 induces its anti-inflammatory effects is by increasing the production of IL-10 [57,58]. Interestingly, this was observed in the current study, as only females displayed significantly higher IL-6 and IL-10 levels on HFD compared to the CD group, as well as when compared to their male counterparts on HFD. This unique finding suggests the IL-6/IL-10 cytokine axis observed only in females may account for their lower weight gain compared to males during the period of DIO, and this strongly suggests sex as an important biological variable to be considered in obesity research. Several studies have reported sexual dimorphism in IL-6 in situations of severe injury, stress, and infection [59,60,61], but not in obesity. Our study is the first to report on sexual dimorphism in IL-6 production during diet-induced obesity. Additionally, it is important to recognize that due to inherent biological variability between individual mouse, chemokine and cytokine production were only detected in some male or female mice, despite them being fed on the same experimental diet. It is, therefore, recommended that future studies should consider increasing the number of animals in each experimental group in order to take into account the existence of biological variability between animals. Overall, we have observed that HFD males displayed a higher concentration of pro-inflammatory cytokines, with no observed differences in the anti-inflammatory cytokine profile, while females displayed notable differences in anti-inflammatory cytokines, like IL-5, IL-6, and IL-10. Thus, it appears that the expression of cytokines in obesity is largely dependent not only on body weight and adiposity but also on sex, which should be considered as an important parameter in future obesity related research. Finally, GM-CSF, TNFα, and IL-12 (p70) were not detected in either CD or HFD, suggesting they do not play a major role in the early stages of obesity development.

## 5. Conclusions

This study provides qualitative and semi-quantitative data demonstrating that the regulation of some plasma analytes, as well as chemokines and cytokines in response to weight gain, are significantly influenced by sex, which potentially may influence the rate and degree to which male and female develop obesity associated comorbidities. This new knowledge may assist in developing suitable intervention strategies for managing the development of obesity associated comorbidities in males and females. 

## Data Availability

The data presented in this study are available in this article and Supplementary materials. All primary data such as weekly weight and food intake measurements, adipocyte sizes and numbers are available on request from the corresponding author.

## Supplementary Materials

The following are available online at https://www.mdpi.com/article/10.3390/biology10080717/s1, Figure S1: Dissection of CD female, A; CD male, B; HFD female, C; and HFD male D; showing the perigonadal white adipose tissue depot, Table S1: Average weight gained, food intake and caloric intake per week by CD females, CD males, HFD females and HFD males, Figure S2: Histology of pancreas harvested from CD female, A; CD males, B; HFD females, C; and HFD males, D. Figure S3: Histology of the thymus harvested from CD females, A and E; CD males, B and F; HFD females, C and G; and HFD males, D and H. Figure S4: Histology of adrenal glands harvested from CD females, A and E; CD males, B and F; HFD females, C and G; and HFD males, D and H. Figure S5: Histology of gastrocnemius muscle (transverse section) harvested from CD females, A; CD males, B; HFD females, C; and HFD males, D. Table S2: Chemokines, anti-inflammatory and pro-inflammatory cytokines measured in the CD and HFD, males and females.
