# Supplementary material for: Sexual Dimorphism in Changes That Occur in Tissues, Organs and Plasma during the Early Stages of Obesity Development
Source: Biology (Basel). 2021 Jul 28;10(8):717. doi: 10.3390/biology10080717 (PMC8389333; doi:10.3390/biology10080717)
Supplement: Supplementary file 1 [file biology-10-00717-s001.zip › biology-1290375-SI.pdf]

Supplementary results

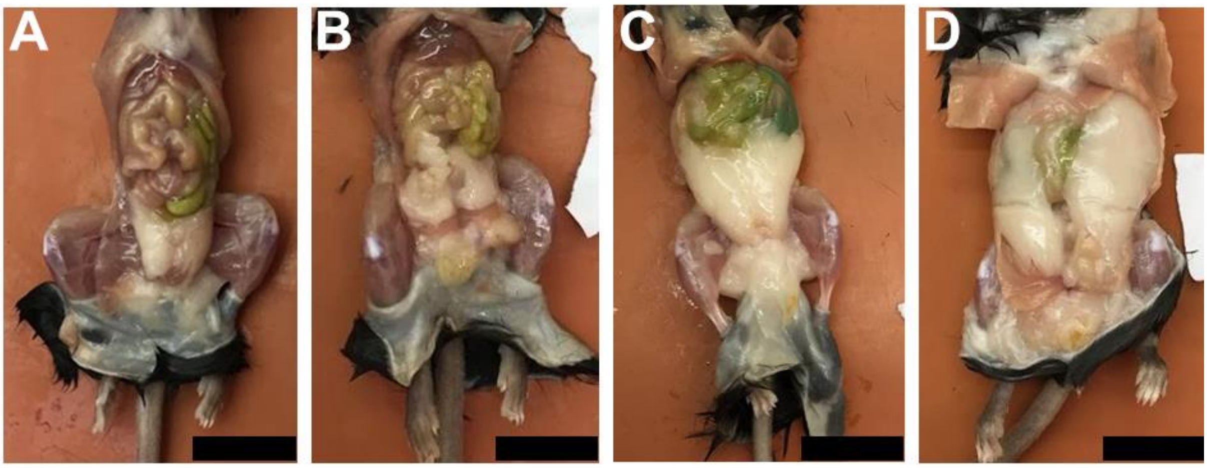

Figure S1: Dissection of an **A**) CD female, **B)** CD male, **C)** HFD female and **D)** HFD male, illustrating the perigonadal white adipose tissue depot. **Scale bar**: 2 cm

Table S1: Average weight gained, food intake and caloric intake per week by CD females, CD males, HFD females and HFD males.

	CD FEMALE	CD MALE	HFD FEMALE	HFD MALE
**Average weight gained/week (g)**	0,16 ± 0,10	0,09 ± 0,24	**0,77 ± 0,17$^{\#}$**	**1,97 ± 0,41$^{¥€}$**
**Average food intake/week (g)**	17,65 ± 0,32	18,44 ± 0,85	17,14 ± 0,84	18,99 ± 1,07
**Average caloric intake/week (kcal)**	66,90 ± 0,73	68,79 ± 2,67	**87,22 ± 4,02$^{\#}$**	**96,90 ± 5,47$^{¥}$**

Mean ± SEM, Females: Week 1-6, 8 and 9 (n=9). Week 7 (n=4). Week 10 and 11 (n=5). Males: Week 1-6, 8 and 9 (n=7). Week 7 (n=4). Week 10 and 11 (n=3). $^{\#}$Significant difference between CD female and HFD female (P<0,05). $^{¥}$Significant difference between CD male and HFD male (P<0,05). $^{€}$Significant difference between HFD female and HFD male (P<0,05).

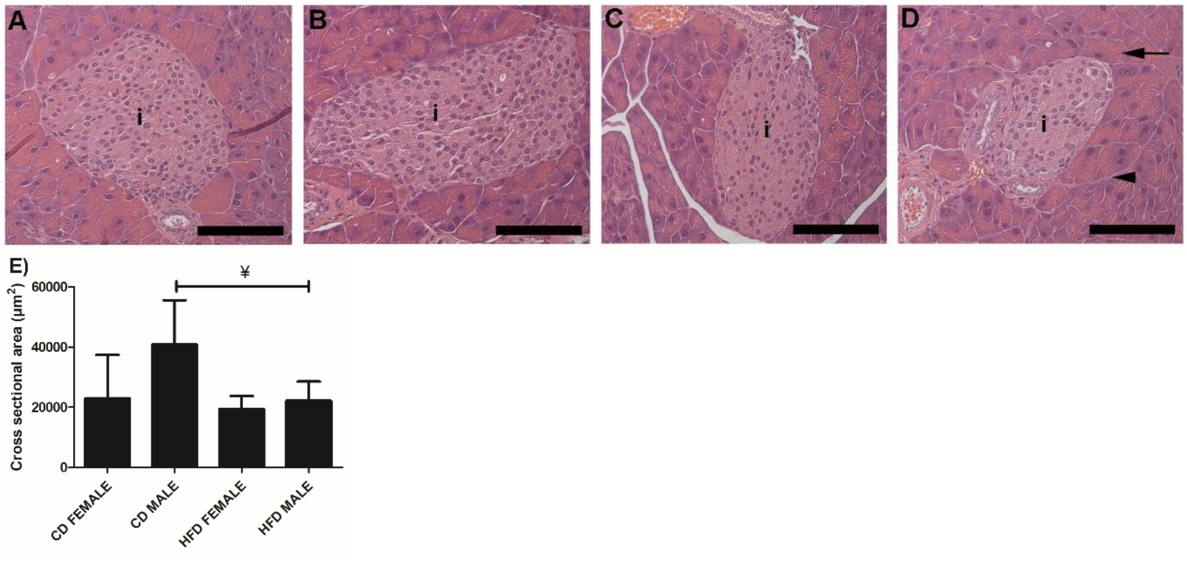

Figure S2: Histology of pancreas harvested from A) CD female, B) CD male, C) HFD female, and D) HFD male. The figures illustrate the endocrine pancreas, islet of Langerhans (i), and the exocrine pancreas. E) Cross sectional area of the islets of Langerhans (n=5). No visible abnormalities were observed in HFD females. In HFD males, enlargement of the acinar cells (black arrow) and partial thickening of the fibrocollagenous septa (arrowhead) were observed, compared to CD males. The islets of Langerhans were significantly smaller in HFD males compared to CD males. Key: ¥ Significant difference between CD male and HFD male. P<0,05. Scale bar: 100 μm.

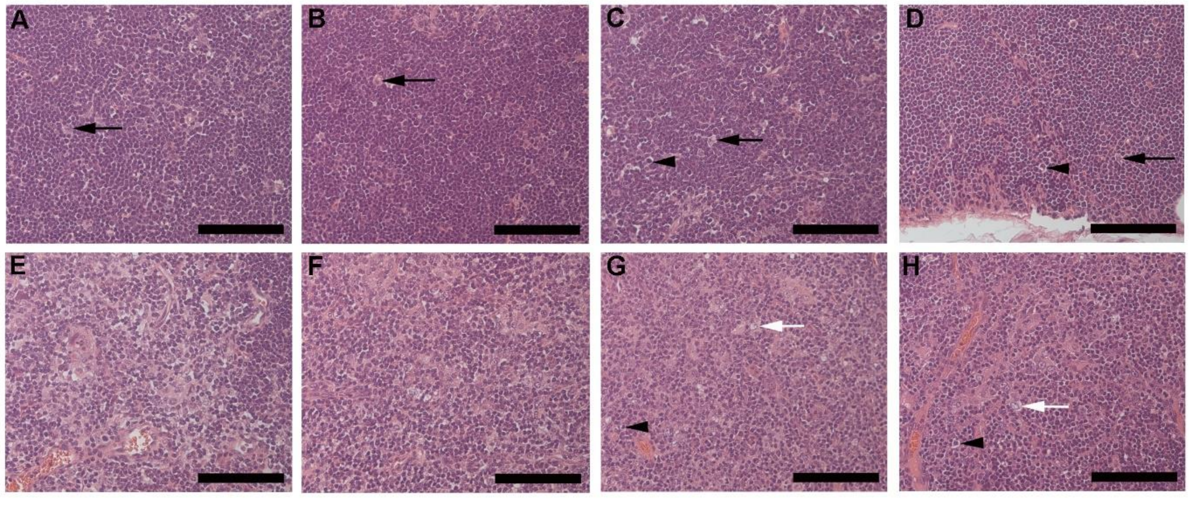

Figure S3: Histology of the thymus harvested from CD females (A and E), CD males (B and F), HFD females (C and G) and HFD males (D and H). Figures S3 A, B, C and D illustrate the cortex of the thymus, composed primarily of lymphocytes. In the cortex, thymic macrophages were visible (black arrow). Figure S3 E, F, G and H illustrate the medulla of the thymus containing lymphocytes and epitheliocytes. Thymic macrophages were observed in all animals, but were more prominent in HFD animals, especially males. In HFD animals, lymphocyte necrosis (arrowhead) and epitheliocyte swelling (white arrow) was evident and was more extensive in males. Scale bar: 100 μm.

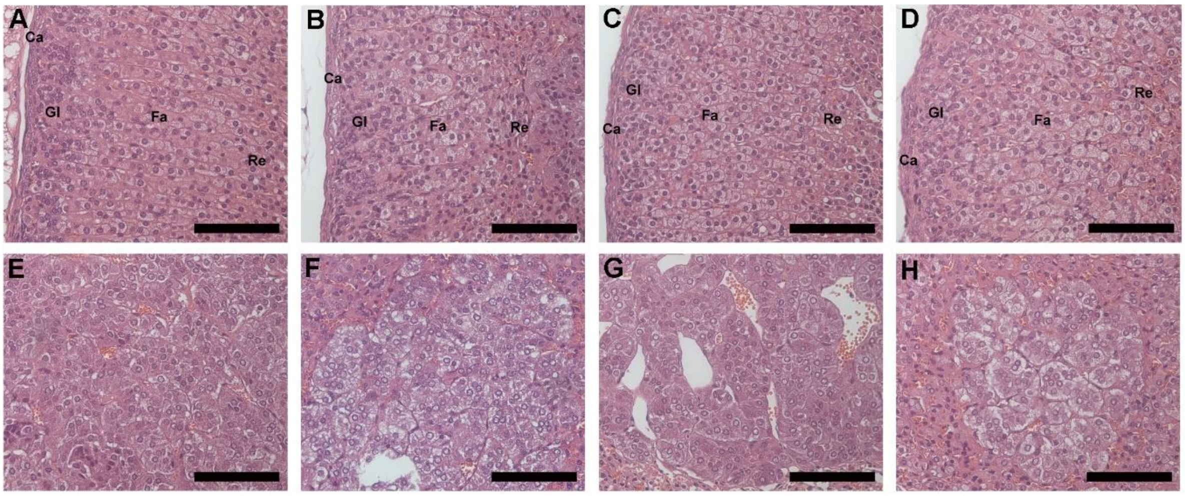

Figure S4: Histology of adrenal glands harvested from CD females (A and E), CD males (B and F), HFD females (C and G) and HFD males (D and H). Figure S4 A, B, C and E show the adrenal cortex composed of capsule (Ca) and three distinct zones, the zona glomerulosa (Gl), zona fasciculata (Fa) and zona reticularis (Re). Figure S4 E, F, G and H illustrate the chromaffin cells making up the adrenal medulla. HFD animals displayed partial enlargement of the zona fasciculata cells, which was more evident in males. Enlargement of chromaffin cells was observed only in HFD males. Scale bar: 100 μm.

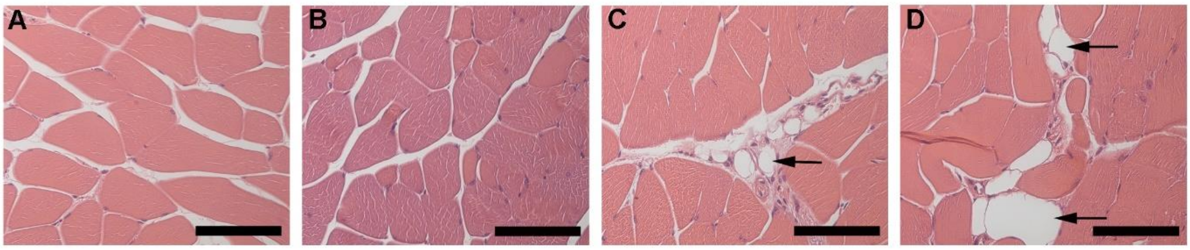

Figure S5: Histology of gastrocnemius muscle (transverse section) harvested from A) CD female, B) CD male, C) HFD female and D) HFD male. In the HFD animals, adipocytes (black arrow) were visible within the perimysium. Larger adipocytes were observed in HFD males compared to HFD females. Scale bar: 100 μm.

Table S2: Chemokines, and anti- and pro-inflammatory cytokines measured in CD and HFD males and females.

	**Concentration (pg/ml)**			
	**CD FEMALE (n=4)**	**CD MALE (n=4)**	**HFD FEMALE (n=5)**	**HFD MALE (n=5)**
**CHEMOKINES**				
**MCP-1**	-	-	-	33,18 ± 12,60 (n=3)
**MIP-1α**	61,44 (n=1)	62,10 ± 17,53 (n=4)	88,63 ± 33,62 (n=4)	88,45 ± 39,13 (n=4)
**MIP-1β**	38,91 ± 18,92 (n=3)	**70,52 ± 13,57 (n=4)***	43,67 ± 15,58 (n=4)	57,17 ± 28,67 (n=3)
**MIP-2**	-	-	63,3 ± 35,45 (n=2)	127,8 ± 64,22 (n=5)
**RANTES**	16,99 ± 3,99 (n=4)	**42,62 ± 17,24 (n=4)***	**26,17 ± 3,547 (n=5)#**	32,57 ± 14,19 (n=5)
**IP-10**	101,2 ± 22,94 (n=4)	157 ± 49,08 (n=4)	**163,2 ± 24,42 (n=5)#**	216,2 ± 114,3 (n=5)
**KC**	49,33 ± 27,59 (n=4)	80,90 ± 41,19 (n=4)	36,12 ± 20,08 (n=5)	**219,3 ± 52,96 (n=4)€¥**
**ANTI-INFLAMMATORY CYTOKINES**				
**G-CSF**	277,60 ± 30,71 (n=4)	**620,90 ± 85,98 (n=3)***	376,00 ± 85,62 (n=5)	**406,3 ± 96,27 (n=5)¥**
**IL-4**	1,87 ± 0,26 (n=4)	2,128 ± 0,14 (n=4)	1,92 ± 0,39 (n=5)	2,13 ± 0,37 (n=5)
**IL-5**	7,79 ± 5,01 (n=4)	5,49 ± 4,35 (n=4)	16,98 ± 12,97 (n=5)	6,62 ± 1,36 (n=5)
**IL-10**	16,86 ± 3,16 (n=4)	**24,52 ± 2,32 (n=4)***	**24,95 ± 5,53 (n=5)#**	26,82 ± 5,38 (n=5)
**IL-13**	13,91 ± 3,11 (n=3)	16,35 ± 2,34 (n=4)	9,97 ± 6,56 (n=5)	12,13 ± 7,56 (n=5)
**PRO-INFLAMMATORY CYTOKINES**				
**GM - CSF**	-	-	-	-
**IFNγ**	2,76 ± 2,23 (n=2)	3,27 ± 3,44 (n=3)	3,25 ± 1,54 (n=4)	0,69 (n=1)
**TNFα**	-	-	-	-
**IL-1α**	31,95 (n=1)	76,53 ± 34,85 (n=2)	113,6 ± 74,8 (n=3)	273 ± 159,80 (n=4)
**IL-1β**	1,98 ± 0,78 (n=2)	1,94 ± 2,38 (n=2)	2,81 ± 0,39 (n=2)	1,76 ± 1,71 (n=3)
**IL-2**	5,83 ± 0,53 (n=4)	6,04 ± 1,07 (n=4)	6,70 ± 0,81 (n=5)	6,47 ± 1,06 (n=5)
**IL-6**	5,54 ± 0,77 (n=4)	**10,60 ± 3,35 (n=3)***	**8,00 ± 1,75 (n=5)#**	11,62 ± 8,42 (n=5)
**IL-7**	3,48 ± 5,00 (n=4)	4,86 ± 5,53 (n=4)	1,70 ± 1,09 (n=4)	2,55 ± 0,94 (n=4)
**IL-9**	209,3 (n=1)	80,79 ± 96,58 (n=4)	206,7 ± 66,52 (n=2)	**284,9 ± 87,09 (n=4)¥**
**IL-12 (p40)**	10,37 ± 4,01 (n=3)	23,1 ± 9,98 (n=4)	23,38 ± 29,75 (n=3)	11,49 ± 9,66 (n=4)
**IL-12 (p70)**	-	-	-	-
**IL-15**	-	-	18,88 (n=1)	49,37 ± 38,29 (n=4)

**IL17**	3,32 ± 1,29 (n=4)	3,94 ± 0,77 (n=4)	3,95 ± 1,30 (n=5)	5,73 ± 2,10 (n=5)

Mean ± SD. *Significant difference between CD females and CD males. # Significant difference between CD females and HFD females. ¥ Significant difference between CD males and HFD males. € Significant difference between HFD females and HFD males. P<0,05.
